# 

**Published:** 1866-12

**Authors:** 


### ORIGINAL CONTRIBUTIONS.

ART. XLII. Cerebro-Spinal Meningitis. By F. R. Payne, M.D., of Marshall, Ill. (Read to the Illinois State Me'ical Society.) 705

ART. XLIII. Bromine and its Compounds. By J. H. Hollister, M.D., and Prof. Chicago Medi al College. (Read to the Illinois State Medical Society.) 71[illegible]

ART. XLIV. On the Therapeutic Uses of Bromide of Ammonium. By Ira Hatch, M.D., Chicago. (Read to the Illinois State Medical Society.) 717

ART. XLV. Carcinoma.—Death from Ulceration and Perforation of the Stomach without Tenderness or Pain. By A. Given, M.D., Louisville, Ky., 722

ART. XLVI. Poisoning by Strychnine. By S. A. McWilliams, A.M., M.D., Chicago, 726

ART. XLVII. Camphorated Oil (*Lin. Camphoræ, U.S.*) and Cot.on, as a Local Application in Erysipelas. By B. H. Cheney, M.D., Joliet, Ill., 72[illegible]

ART. XLVIII. A Few Practical Suggestions on the Management of Chronic Ulcers. By R. Dexter, M.D., Chicago, 731

ART. XLIX. The Relations of Cancer and Consumption to Climate in the United States. By E. Andrews, M.D., Prof. of Sur_ery, 737

### PROCEEDINGS OF SOCIETIES.

Proceedings of the Morgan County Medical Society, 740
Proceedings of the Esculapian Society of the Wabash Valley, 743

### THE CLINIQUE.

Case of Cholera—Unusually Protracted Suppression of Urine—Recovery. By N. S. Davis, M.D., Prof. Practical and Clinical Medicine, etc., 746

### BOOK NOTICES.

A Manual of Ascultation and Percussion. By M. Barth and M. Henri Roger. Translated from the sixth French e lition, 751
A Manual of Medical Jurisprudence. By Alfred Swayne Taylor, M.D., 751
A Practical Treatise on Diseases of the Skin. By J. Moore Neligan, M.D., 751
An Introduction to Practical Chemistry, Including Analysis. By John E Bowman, F.C.S., etc. Edited by Charles L. Bloxam, F.C.S., etc., 752

### EDITORIAL.

Clinical Instruction on Diseases of the Eye and Ear, 752
Cancer Curers, 752
Chicago Medical Society, 753
Close of the Volume, 753
Prize Questions, 753
Statistics of Cook County Hospital, 754
Announcement of the Physician's Pocket Record, for 30 Patients, 755
Obituary.—Surgeon and Brevet-Brigadier-General C. S. Tripler, U.S.A., 756
Prize Essays, 757
Guaco as a Remedy for Cholera, 759
Mortality for the Month of October, 759
Mortality for the Month of November, 760
Public Health.—Comparison of Cities, 761
Swinging as a Remedy, 762

